# Determination of Somatic Mutations and Tumor Mutation Burden in Plasma by CAPP-Seq during Afatinib Treatment in NSCLC Patients Resistance to Osimertinib

**DOI:** 10.1038/s41598-020-57624-4

**Published:** 2020-01-20

**Authors:** Hidenobu Ishii, Koichi Azuma, Kazuko Sakai, Yoshiko Naito, Norikazu Matsuo, Takaaki Tokito, Kazuhiko Yamada, Tomoaki Hoshino, Kazuto Nishio

**Affiliations:** 1 Division of Respirology, Neurology, and Rheumatology, Department of Internal Medicine, Kurume University School of Medicine, Kurume, Fukuoka Japan; 2 Department of Genome Biology, Kinki University Faculty of Medicine, Osaka, Japan; 3 Department of Respiratory Medicine, Shin Koga Hospital, Kurume, Japan

**Keywords:** Non-small-cell lung cancer, Non-small-cell lung cancer, Cancer genomics, Cancer genomics

## Abstract

Third-generation epidermal growth factor receptor (EGFR) tyrosine kinase inhibitors (TKIs) were developed to target the *EGFR* T790M resistance mutation in non-small cell lung cancer (NSCLC) patients resistant to first- or second-generation EGFR-TKIs. To investigate the efficacy of afatinib treatment for *EGFR* T790M-positive NSCLC patients showing resistance to osimertinib and alterations in somatic mutations and tumor mutation burden (TMB) in plasma circulating tumor DNA (ctDNA) during afatinib treatment, we conducted a prospective study using Cancer Personalized Profiling by deep Sequencing (CAPP-Seq). Nine NSCLC patients with *EGFR* T790M mutation who showed resistance to third-generation EGFR-TKIs were enrolled in this study and treated with afatinib. Plasma samples were collected before treatment, 4 weeks after treatment, and at disease progression. The mutation profile and TMB in plasma ctDNA were analyzed by CAPP-Seq. The objective response rate and median progression-free survival associated with afatinib were 0% and 2.0 months, respectively. The C797S mutation-mediated resistance to osimertinib was observed in one patient and following afatinib treatment in two patients; the C797S mutations occurred in the same allele as the T790M mutation. After afatinib treatment, afatinib-sensitive mutant alleles, such as ERBB2, and TMB decreased. We have demonstrated that detection of mutant allele frequency and TMB of ctDNA by CAPP-Seq could help determine the effectiveness of and resistance to afatinib. Although afatinib monotherapy for T790M-positive NSCLC resistant to osimertinib was less effective, the action for multiclonal mutant alleles and TMB might contribute to further treatment strategy.

## Introduction

Non-small-cell lung cancer (NSCLC) is the most common cause of cancer-related death worldwide^1^. The epidermal growth factor receptor (*EGFR*)-activating mutations have been identified as a definitive predictive marker of the favorable efficacy for treatment with EGFR tyrosine kinase inhibitors (EGFR-TKIs) in NSCLC patients^2–5^. Although NSCLC patients harboring *EGFR* mutations generally achieve clinical benefits from EGFR-TKI treatment, most patients show the development of resistance to EGFR-TKIs after approximately 1 year^2–5^. Osimertinib is designed to target the *EGFR* T790M resistance mutation, which is the most frequently event responsible for resistance to initial EGFR-TKI treatment in *EGFR*-mutated NSCLC patients, and patients with this mutation show a better response to osimertinib than to cytotoxic chemotherapy^6^. However, the patients also eventually acquire resistance to osimertinib. Recently, the mechanisms of acquired resistance to osimertinib have been elucidated. Of them, the replacement of cysteine with serine at codon 797 (C797S) has been reported as one of the mechanisms for resistance to osimertinib^7–9^. Although cell lines with the C797S mutation were sensitive to quinazoline-based EGFR inhibitors, including gefitinib or afatinib^10^, the clinical utility of gefitinib or afatinib remains unclear for next-line treatment after resistance to osimertinib has been acquired by the cells.

To research the efficacy of afatinib treatment in *EGFR* T790M-positive NSCLC patients showing resistance to osimertinib and alterations in somatic mutations and tumor mutation burden (TMB) in plasma circulating tumor DNA (ctDNA) during afatinib treatment, we conducted a prospective study using Cancer Personalized Profiling by deep Sequencing (CAPP-Seq).

## Materials and Methods

### Study design and eligibility

**Figure 1:**
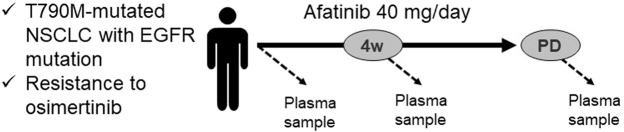
The schema of this study. Plasma samples were collected before and 4 weeks after afatinib treatment, and at the development of disease progression.

The study schema is shown in Fig. 1. Eligible patients were aged 20 years or more, had histologically or cytologically confirmed adenocarcinoma of the lung with an *EGFR* T790M mutation, and were previously treated with osimertinib, which resulted in acquired resistance. Additional major inclusion criteria were measurable lesion according to the Response Evaluation Criteria in Solid Tumors (RECIST v.1.1), an Eastern Cooperative Oncology Group Performance Status (PS) of 0 to 2, and adequate organ function. The exclusion criteria were pleural or pericardial effusion required drainage, metastatic brain tumor requiring treatment, active multiple primary cancer, and a medical history of interstitial lung disease. We conducted the study in accordance with the provisions of the Declaration of Helsinki. All experimental protocols were approved by the Institutional Review Board of Kurume University Hospital (IRB No. 16067) and was registered with the University Hospital Medical Information Network (UMIN) in Japan (number UMIN 000025126). Written informed consent was obtained from all participants.

### Sample collection

For plasma samples, 14 mL of peripheral blood was collected in EDTA-coated tubes before and 4 weeks after afatinib treatment, and upon disease progression. Plasma was separated by centrifugation at 1000 rpm for 15 min within 2 h of sample collection and stored at −80 °C until DNA extraction. Plasma ctDNA was purified using an AVENIO cfDNA isolation kit (Roche Diagnostics), and the quality and quantity of the DNA were verified using the NanoDrop 2000 device (Thermo Scientific) and PicoGreen dsDNA assay kit (Life Technologies) according to previous study^11^. The extracted ctDNA was stored at −80 °C until the analysis.

### Mutation profile and TMB analyses

We analyzed the mutation profile and TMB as previous study^11^; A maximum of 50 ng of DNA was used for the CAPP-Seq ctDNA analyses using the AVENIO ctDNA surveillance kit (Roche Diagnostics, 197 genes). The purified libraries were pooled and sequenced on an Illumina NextSeq. 500 sequencing system (Illumina) using the 300-cycle high output kit. Variants were called with the AVENIO ctDNA Analysis Software (Roche Diagnostics), which includes bioinformatics methods from CAPP-Seq2^12^ and integrated digital error suppression^13^. Germline mutations were excluded with the use of the Human Genetic Variation Database (http://www.genome.med.kyoto-u.ac.jp/SnpDB) and the ExAC database.

## Results

### Patient characteristics

Between December 2016 and January 2018, nine patients were enrolled in this prospective study and treated with afatinib. The characteristics of enrolled patients are shown in Table 1. Seven patients were female and two were male. Four patients had an E746-A750 deletion in exon 19, and five had an L858R point mutation in exon 21. No *EGFR* minor mutations were detected in all patients. Afatinib treatment was provided as third-line chemotherapy in three patients, fourth-line chemotherapy in four, and sixth-line chemotherapy in two. The median progression-free survival to initial osimertinib treatment was 8.2 months.

**Table 1:** Patient characteristics.

Patient	Age (years)	Gender	Smoking	Histology	*EGFR* mutation at initial diagnosis	Line of treatment
KU-01	65	Female	Never	adeno	E746-A750 del	3rd
KU-02	71	Male	Never	adeno	E746-A750 del	6th
KU-03	71	Female	Never	adeno	L858R	6th
KU-04	68	Female	Never	adeno	L858R	4th
KU-05	75	Female	Never	adeno	L858R	3rd
KU-06	78	Male	Former	adeno	L858R	4th
KU-07	81	Female	Never	adeno	E746-A750 del	3rd
KU-08	58	Female	Former	adeno	E746-A750 del	4th
KU-09	89	Female	Never	adeno	L858R	4th

EGFR, epidermal growth factor receptor.

### Efficacy of afatinib treatment

All patients received an initial afatinib dose of 40 mg/day. The efficacy of afatinib treatment in each patient is shown in Table 2. At 4 weeks after afatinib therapy, four patients indicated disease progression and five patients exhibited stable disease. The median progression-free survival time was 2.0 months.

**Table 2:** Efficay and alterations in *EGFR* mutations of afatinib treatment.

Patient	*EGFR* mutations in ctDNA before Afatinib	Response to Afatinib	PFS (months)	*EGFR* mutations in ctDNA progression after Afatinib
KU-01	E746-A750 del, T790M	SD	1.8	E746-A750 del, T790M, C797S
KU-02	E746-A750 del, T790M	SD	3.4	E746-A750 del, T790M, C797S
KU-03	L858R	SD	2.5	L858R, T790M
KU-04	L858R	SD	1.5	L858R
KU-05	L858R	PD	0.9	L858R
KU-06	L858R, T790M	PD	0.9	L858R, T790M
KU-07	T790M, C797S	PD	0.9	T790M, C797S
KU-08	absent	PD	2.2	absent
KU-09	L858R	PD	2.0	L858R

EGFR, epidermal growth factor receptor; ctDNA, circulating tumor DNA; PFS, progression-free survival; SD, stable disease; PD, progressive disease.

### Mutation profile at resistance to osimertinib

A total of 36 somatic mutations or amplifications were detected in plasma cfDNA before afatinib treatment: *EGFR*-activating mutations in eight patients; TP53 mutations in six patients; T790M mutations in four patients; *PIK3CA* and *BRAF* mutations and MET amplification in three patients each; *CTNNB1* and *ERBB2* mutations in two patients each; and *EGFR* C797S and SMAD4 mutations, *EGFR* minor mutation, *KRAS* mutation and adenomatous polyposis coli (*APC*) mutation in one patient each.

### Monitoring of somatic mutations and TMB during afatinib treatment

**Figure 2:**
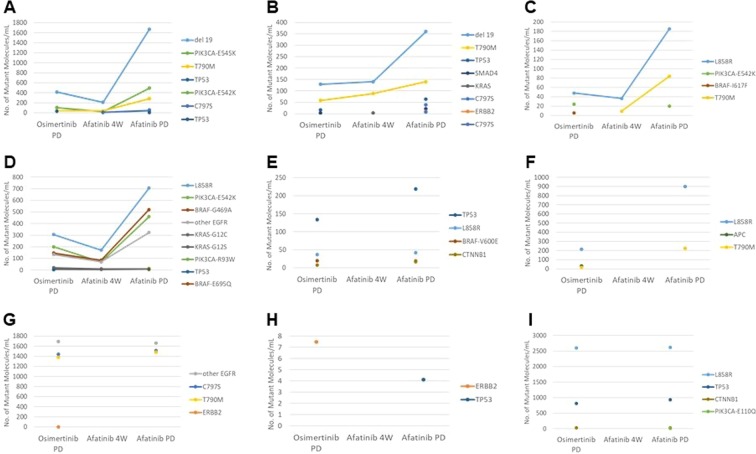
Analysis of somatic mutations in plasma circulating tumor DNA by CAPP-Seq. Alterations of the mutant allele in nine patients before and 4 weeks after afatinib treatment, and at the development of disease progression (**A–I**).

**Figure 3:**
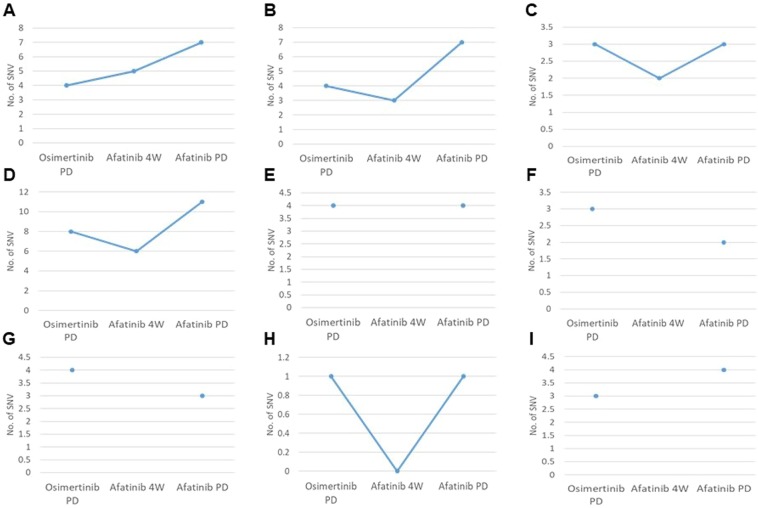
Analysis of the tumor mutation burden in plasma circulating tumor DNA by CAPP-Seq. Tumor mutation burden in nine patients before and 4 weeks after afatinib treatment, and at the development of disease progression (**A**–**I**).

The alterations in mutant allele frequencies with afatinib treatment in each patient are shown in Fig. 2 and alterations in *EGFR* mutations are summarized in Table 2. In patients who maintained the T790M mutation after showing resistance to osimertinib, the number of mutant T790M molecules increased during afatinib treatment. One patient showed the appearance of T790M mutation following afatinib treatment. The ERBB2 mutation was confirmed with resistance to osimertinib in two patients. Although afatinib treatment was not effective in both patients, *ERBB2* mutation disappeared almost completely after afatinib treatment. *EGFR* C797S mutation was observed with resistance to osimertinib in one patient and following afatinib treatment in two patients. In all patients, C797S mutations occurred in the same allele as the T790M mutation. Alterations of TMB by afatinib treatment are also shown in Fig. 3. In patients who achieved stable disease at 4 weeks of afatinib treatment, TMB generally decreased once and re-increased at disease progression.

## Discussion

In this study, we investigated the mechanisms of acquired resistance to osimertinib and the alterations in mutant allele frequency and TMB during subsequent afatinib treatment by CAPP-Seq in NSCLC patients with *EGFR* T790M mutation.

The C797S mutation was detected with resistance to osimertinib in one patient. This case showed no response to the subsequent afatinib treatment. Furthermore, two patients exhibited the C797S mutation during afatinib treatment. The resistance pattern of C797S is categorized into two types according to the allocation pattern of C797S and T790M mutations^14^. The preclinical experiment showed C797S and T790M exist on separate alleles (in trans pattern), the resistant cells show sensitivity to afatinib, however, when C797S and T790M coexist on the same allele (in cis pattern), none of the available EGFR-TKIs is effective^14^. In all three cases exhibited the C797S mutation, the C797S mutation was identified in cis pattern. Consistent with preclinical findings, afatinib treatment seemed to be less effective for these patients.

Among the patients, five patients exhibited the loss of T790M mutation before afatinib treatment. Therefore all patients were previously treated with osimertinib at the time of enrollment, it was considered that loss of T790M mutation in these patients was occurred by osimertinib treatment as previous report^15^. While, four patients showed a remaining T790M mutation with resistance to osimertinib. These cases exhibited an increase in the T790M mutant allele frequency during afatinib treatment. Furthermore, one patient exhibited re-emergence of T790M mutation at the development of disease progression after afatinib treatment. Similarly, the patients with the *TP53* mutation showed an increase in allele frequency with afatinib treatment. The T790M mutation occurs as a result of the resistance mechanism to afatinib treatment in *EGFR*-mutated NSCLC, and mutations in the tumor suppressor gene *TP53*, which have been associated with tumor progression and poor prognosis in various malignancies^16–18^, may play a role in the resistance to EGFR-TKIs in *EGFR*-mutated NSCLC^19^.

Two patients exhibited ERBB2 mutant alleles with resistance to osimertinib, and both cases showed the disappearance of the ERBB2 mutant allele after afatinib treatment. ERBB2 amplification is well known as one of the mechanisms of resistance to EGFR-TKIs, including third-generation EGFR-TKIs^20,21^. Afatinib, which is an irreversible tyrosine kinase inhibitor, binds to the kinase domains of *EGFR*, *ERBB2*, and *ERBB4*^22^. The inhibitory effect of afatinib against ERBB2 might have provided a favorable effect in this patient.

**Figure 4:**
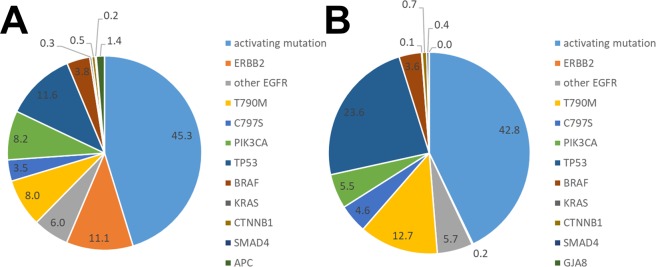
The mutant allele frequency before afatinib treatment (**A**) and after disease progression (**B**) in all patients.

In this study, we also investigated the alterations in TMB during afatinib treatment. In patients with stable disease at 4 weeks after treatment, TMB tended to decline once and then increased in association with disease progression. The mutant allele frequencies before afatinib treatment and after disease progression in all patients are summarized in Fig. 4. Various mutant alleles showed varying frequencies at resistance to osimertinib. The resistance mechanisms post-osimertinib in plasma samples of patients participating in the AURA 3 trial were reported recently^23^. Consistent with our results, that study showed a huge variety of mechanisms, including C797S mutations, MET amplification, *HER2* amplification, *PIK3CA* mutation, and *BRAF* V600E mutation. However, the subsequent treatment after resistance to osimertinib is not established. Our study suggested that afatinib treatment might induce the selection of mutant clones, and that possibly led to the decrease of TMB. However, the proportions of *EGFR* mutations including T790M mutation increased among the mutant alleles with the disease progression after afatinib treatment. The mechanisms underlying the resistance to osimertinib were heterogeneous and it might be important to control the T790M mutation in treatment following osimertinib. Therefore, our findings indicate the possibility of using combination therapy with afatinib and osimertinib or cytotoxic chemotherapies. We are currently conducting a phase I study with afatinib plus osimertinib in *EGFR*-mutated NSCLC patients previously treated with osimertinib (UMIN-CTR: 000031501).

Our study had several limitations. A major weakness is that the number of patients studied was relatively small. Second, no patients showed tumor shrinkage with afatinib monotherapy. None of the patients exhibited C797S mutation in trans, which could respond to afatinib. Further studies are warranted to investigate the clinical efficacy of afatinib treatment for patients with a C797S mutation in trans in larger samples.

## Conclusions

We have demonstrated that detection of the mutant allele frequency and the TMB of ctDNA by CAPP-Seq could allow monitoring of the effectiveness and resistance to afatinib. Although afatinib showed no clinical efficacy after resistance to osimertinib in patients with *EGFR* T790M mutation, afatinib treatment might reduce the number of afatinib-sensitive mutant alleles and the TMB. These findings suggest a possible beneficial effect of afatinib-based combination therapy, such as afatinib plus osimertinib.
